# C-857-06. Ethical Challenges of Bioprinted and Lab-grown Skin Substitutes in Burn Care

**DOI:** 10.1093/jbcr/irag033.106

**Published:** 2026-04-06

**Authors:** Joshua Khorsandi, Liahm Blank, Abu-Bakr Ahmed, Samir Alkhouri, Demitri Franzoni

**Affiliations:** Kirk Kerkorian School of Medicine at University of Nevada, Las Vegas, NV; Kirk Kerkorian School of Medicine at University of Nevada, Las Vegas, NV; Kirk Kerkorian School of Medicine at University of Nevada, Las Vegas, NV; Kirk Kerkorian School of Medicine at University of Nevada, Las Vegas, NV; Department of Plastic and Reconstructive Surgery, University of Nevada, Las Vegas, NV

## Abstract

**Introduction:**

Advances in burn care are increasingly driven by biotechnologies such as 3D bioprinting and lab-grown skin substitutes. These therapies hold transformative potential for improving outcomes in patients with severe burns by reducing reliance on cadaveric grafts and donor sites. Yet, alongside these innovations arise profound ethical questions. Issues of ownership, patient consent, intellectual property, and distributive justice challenge conventional bioethics frameworks. Specifically, questions emerge regarding who owns bioprinted or lab-derived tissue, whether patients should retain rights when their cells are commercialized, and how equitable access can be ensured if such grafts become prohibitively expensive. Despite the clinical momentum of these technologies, ethical discourse in the burn care literature remains limited.

**Methods:**

A structured literature review was conducted across PubMed, Embase, and Web of Science from 2010–2025, using search terms including “skin substitutes,” “bioprinting,” “ownership,” “consent,” “intellectual property,” and “burn ethics.” Articles were screened for relevance to ethical, legal, and distributive justice concerns in regenerative medicine and burn care. References were cross-checked to identify secondary literature and emerging perspectives.

**Results:**

The review revealed that current publications primarily address technical efficacy, biocompatibility, and safety, while ethical considerations appear sporadically in commentary pieces rather than empirical studies. Themes identified include: (1) the ambiguous status of patient-derived cells once incorporated into bioprinted grafts, (2) inconsistent consent frameworks regarding commercialization of biospecimens, (3) intellectual property disputes over engineered tissues, and (4) the risk of inequitable access if novel grafts enter markets as premium-priced biotech products. The literature reveals a notable gap in systematic analysis of distributive justice and patient autonomy in this context.

**Conclusions:**

While skin substitutes and 3D bioprinting herald a new era in burn care, ethical frameworks have not kept pace with clinical innovation. Ownership rights, commercialization of patient cells, and access disparities remain unresolved and underexplored.

**Applicability to Practice:**

Burn surgeons, researchers, and policymakers must proactively engage in ethical discourse before widespread clinical adoption. Establishing clear consent processes, transparent ownership frameworks, and equitable pricing models is essential to ensure that bioprinting advances not only technical outcomes but also justice in patient care.

